# Post-mortem computed tomography in adult non-suspicious death investigation—evaluation of an NHS based service

**DOI:** 10.1259/bjro.20190017

**Published:** 2019-07-26

**Authors:** Claire Robinson, Aparna Deshpande, Cathy Richards, Guy Rutty, Catherine Mason, Bruno Morgan

**Affiliations:** 1 Department of Imaging, University Hospitals of Leicester NHS Trust, Leicester, England; 2 Department of Histopathology, University Hospitals of Leicester NHS Trust, Leicester, England; 3 East Midlands Forensic Pathology Unit, University of Leicester, England; 4 The Coroner’s Court, Town Hall, Town Hall Square, Leicester, England; 5 University of Leicester Imaging Department, University Hospitals of Leicester, Leicester Royal Infirmary, Leicester,

## Abstract

**Objective:**

Post-mortem CT (PMCT) can replace autopsy in many cases of non-suspicious death. A purely NHS-based service to replace autopsy with PMCT was launched, with the cost met by the family from 2015 to 2017, and subsequently “free at the point of delivery” after local authority funding was secured. The aim of the service was to improve the experience for the families. This report describes and evaluates the service against local standards of (1) less than four day turn around, (2) cause of death given in >90% and (3) less than 10% require autopsy.

**Methods:**

A retrospective review of reports, records and emails was undertaken to collate demographics, times of different stages of the process, the outcome and comments from service users.

**Results:**

Between July 2015 and July 2018, 279 patients had PMCT scans, 67 (24.0%) in the family-funded service and 212 (76%) in the current service. 97.1% (*n* = 271/279) of cases had the radiology report issued by day 3 (96.8% *vs* 98.6% for the family funded and local authority-funded services respectively). A cause of death was given in 97.2% of scans. 2.8% of patients required autopsy. Feedback from families, coroner’s officers and undertakers has been overwhelmingly positive.

**Conclusion:**

The services exceeded local standards and met the needs of the Coroner and the families based on the feedback received. This model could be employed for similar services, but the change to the logistics and financial structures required to initiate such services remains a significant hurdle.

**Advances in knowledge:**

This is the first report of a fully NHS-based PMCT service.

## Introduction

Over 85,000 autopsies are carried out each year in England and Wales. The majority of these cases do not result in inquest and are found to be natural non-suspicious deaths.^1^ Published autopsy-controlled studies have shown that post-mortem CT (PMCT) could replace autopsy in many of these cases, including many non-suspicious traumatic deaths.^2–5^

In 2015, a local PMCT service was offered for cases of non-suspicious assumed natural death to diagnose the cause of death, exclude unnatural causes and avoid autopsy where possible. This service was provided for the Coroner for Leicester City and South Leicestershire whose jurisdiction covers urban, rural and hospital deaths. The PMCT scan was at the expense of the next-of-kin because local authority (LA) funding could not be secured (family-funded service).

In 2017, management and financial changes were made to allow us to offer this PMCT service within the funding available from the LA for mortuary and registration services. The family-funded service was therefore withdrawn as it was no longer required. The current service (LA funded service) is therefore now “free at the point of delivery” and is the first PMCT service operating entirely within the NHS.

This report describes and evaluates this service: the time taken, the efficacy of the triage system and how often and why autopsies were required. Areas for improvement are also discussed, including practical aspects of the service and why people chose not to use the service having made an enquiry.

The objective of the service is to provide a minimally invasive imaging alternative to autopsy without delaying the release of the body to the next-of-kin. The ultimate aim is to improve the experience for the families of the deceased during the investigation of the death of their loved one.

The service standards were:

- PMCT scan to be performed and reported by day 3 from referral (to allow autopsy by day four if required)
- A cause of death (CoD) will be given in >90% of cases
- Less than 10% of cases will require autopsy after PMCT.

### The service processes

The family-funded PMCT service was offered to the next of kin for all cases referred to the Coroner for Leicester City and South Leicestershire unless the Coroner specifically required an invasive autopsy examination. The Coroner’s Officers explained the process, time scale and the cost involved. If the family chose to request PMCT, they were given the contact details of the radiology department. On contact, the family were further informed of the process and specifically told that in some circumstances PMCT may not provide a CoD and that an invasive autopsy may still be required. This information was supplemented by an information leaflet. If the family requested the service, a pathologist vetted the case to determine if a PMCT scan would be likely to provide the CoD on the “balance of probabilities,” the level of evidence required for the Coroner’s court (Table 1). An external examination of the body was also completed by a pathologist and undertaken prior to PMCT in all cases to ensure there were no signs to suggest an unnatural or suspicious death.

**Table 1.** Triage considerations: circumstances where an autopsy may be required in addition to PMCT

Triage Considerations
Concerns about previous medical care in hospital or any other residential facility
Police or any agency (*e.g.* Health and Safety Executive) were conducting an ongoing investigation into the circumstances of death
Toxicology or histology likely to be required to give a CoD
Infectious diseases (*e.g.* HIV, TB) that may preclude the use of angiography and/or ventilation during the PMCT scan
Medical intervention or trauma to the neck precluding the body preparation required for angiography and /or ventilation
Any unexplained significant trauma in the background information or found on external examination
Patient size and weight above CT scanner limits
Signs of advanced tissue decomposition/autolysis

CoD, cause of death; PMCT, post-mortem CT.

Families were advised if PMCT was felt unlikely to give the CoD without subsequent autopsy. Further exclusions for the PMCT service were if the body size exceeded the bore of the CT scanner (normally 70 cm) and suspicion of a transmissible disease that preventing body preparation for PMCTA and VPMCT. Families were also warned that an autopsy may be required if the PMCT service failed to reach a cause of death.

The opinion of the pathologist was relayed to the family via the service coordinator or the Coroner’s Office. The service offered was specifically to perform a PMCT scan (with radiological interpretation by a consultant radiologist experienced in PMCT), which would then be likely to provide enough information to make invasive autopsy unnecessary. This is distinct to a service to provide a “cause of death,” which would not always be possible based on PMCT alone. PMCT scan was therefore not refused if the pathologist advised that PMCT alone may not provide the CoD and the family still wanted the scan, but the system ensured that the families understood that subsequent autopsy was more likely. When the family was fully informed and agreed to proceed with the scan, payment of £400 + 20% VAT tax was taken. The body was then prepared for the scan by the mortuary technicians.

**Figure 1.**
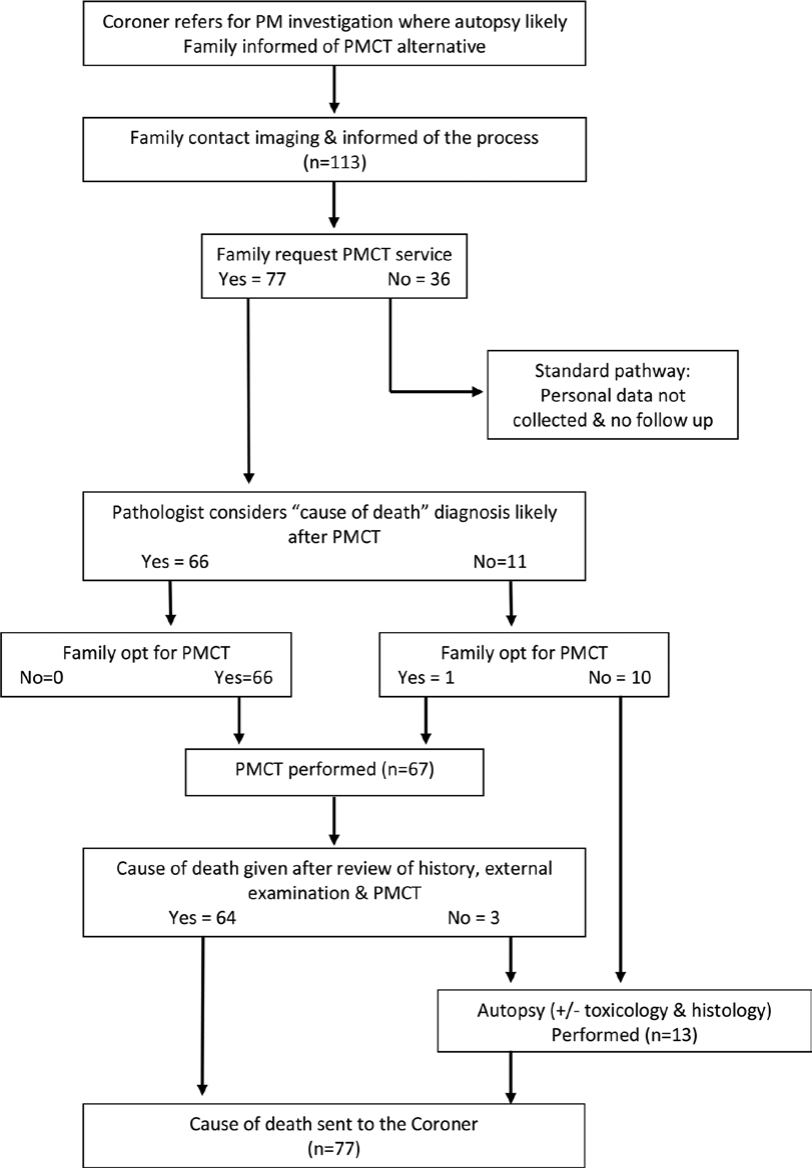
STARD diagram of the family funded service. PMCT,post-mortem CT.

Scans were performed outside standard clinical working hours following our standard PMCT protocol with the addition of targeted coronary angiography (PMCTA) and pulmonary insufflation/ventilation (VPMCT) as described previously.^6–9^ All scans were completed by radiographers specifically trained in PMCT with at least one years’ experience. Support by telephone was provided by the lead radiographer or radiologist. All scans were reported by a radiologist with access to the case history and any important external examination findings. The radiologists were all consultant grade, had undergone training in PMCT and mentoring for their first 3 months in practice. The referring pathologist then reviewed all information and decided whether a CoD could be given or whether further information was required. This was after discussion with the radiologist if necessary. The pathologists were all familiar with the use of PMCT as an alternative to the internal exam at autopsy and were aware of the strengths and weaknesses of the radiology report. Pathologists and radiologists had the opportunity to discuss the findings if required. The family were informed of the CoD by the Coroner’s Office, who also dealt with any further questions from the family (Figure 1).

To register a death in England and Wales a medical certificate of cause of death (MCCD) is required. This is formulated in two parts. Part 1 has three sections and concerns the disease or condition leading directly to death (1a), with 1b and 1c available to identify any disease or condition that has directly led to 1a or 1b respectively. Part 2 may be omitted and lists other significant conditions contributing to death, but not related to the disease or condition causing it. For example, 1a haemopericardium due to ruptured left ventricle, 1b acute myocardial infarction, 1c coronary artery atherosclerosis, 2: essential hypertension and Type II diabetes mellitus.

**Figure 2.**
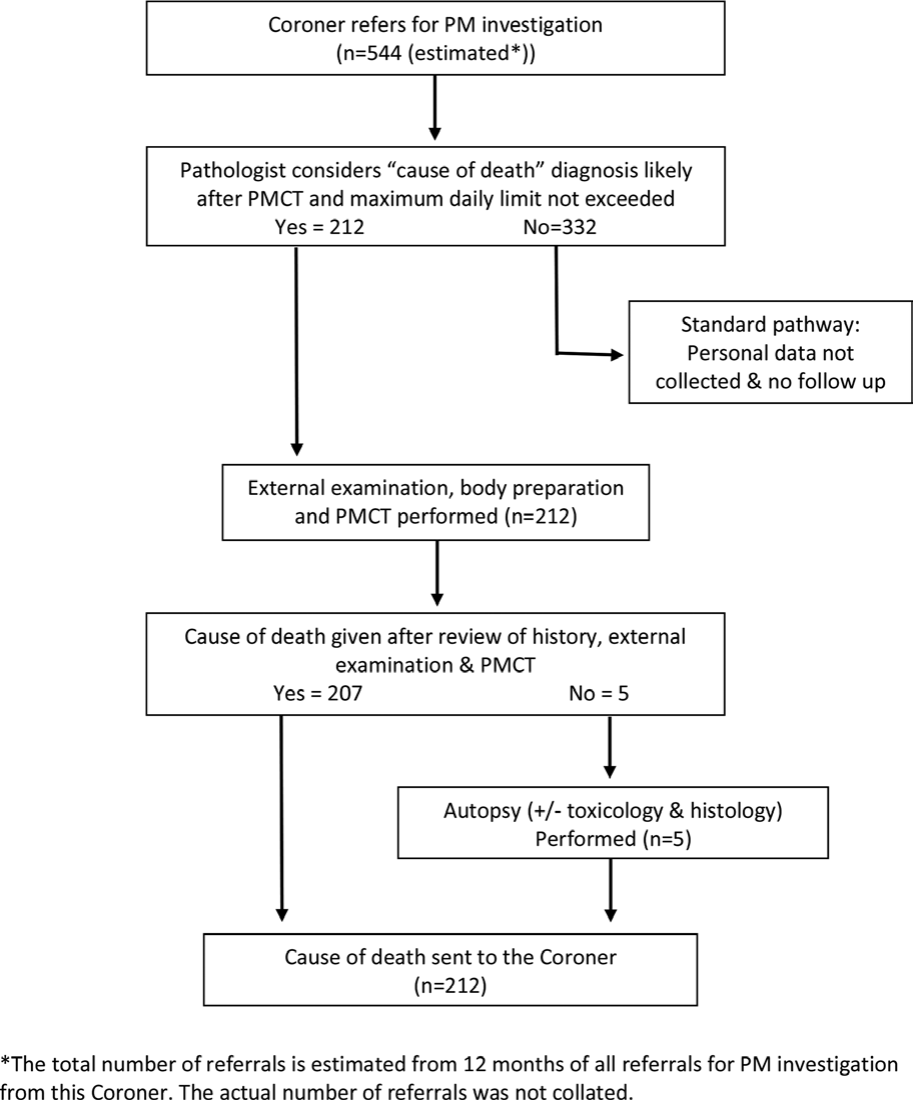
STARD diagram of the local authority funded service. PMCT,post-mortem CT.

The local strategy was always that this family-funded service was a preliminary stage to providing a “free at the point of delivery” PMCT service, funded by the LA under the authority of the Coroner, as for invasive autopsies. The first stage of this was achieved in 2017 due to two key developments: firstly, a financial agreement between pathology, radiology, the Coroner and the LA and secondly, extending the life of an NHS clinical scanner, which was to be removed after replacement in a different area. This created the current LA-funded service performed within the NHS running jointly with the routine autopsy service for HM Coroner referrals. This created two simplifications: firstly, there was no longer a need to delay the process to fully inform families and take payment and secondly, scans could be performed during the working day in greater numbers providing cost savings. This simplified the process. A duty pathologist vetted all cases and determined those suitable for PMCT each morning. The pathologists completed a referral form authorising the mortuary staff to refer to scan and complete the body preparation. On receiving the referrals, the radiology department arranged the scans starting at 14:00 each day. A team of NHS radiologists, locally trained in PMCT, were informed as soon as the scans had been completed, given the background details and informed of any significant findings on the external examination performed by the pathologist. They aimed to review the scans either the same day or the next morning, depending on clinical workflows. The PMCT report was then sent to the pathologist, with the opportunity for discussion if required. The pathologist decided whether an invasive autopsy was required or not and if not, provided the CoD to the Coroner (Figure 2).

All study participants were cannulated within a Human Tissue Authority licensed mortuary, principally using a 16 Fr silicone-coated male urinary catheter (Bardia Foley catheter) inserted into the ascending aorta, just above the aortic valve, via the left common carotid artery by means of a cut-down procedure. PMCT was undertaken using a Toshiba Aquilion 64 slice scanner. Pre-contrast scans were performed in three overlapping blocks of “head & neck,” “chest, abdomen and pelvis” and “pelvis and legs.” Contrast runs through the heart used five separate sequences with 300 ml air for the first three sequences, followed by two sequences of 150 ml Urografin^®^ 150 mg ml^−1^ (Bayer Healthcare, positive contrast) diluted 1:10. Scans were performed dynamically during injection using a Medrad Stellant dual head pump injector system (Medrad UK Ltd, UK) with air at 6 ml/s and scan delay and contrast at 3 ml/s and scan delay 43 s.

## Method

The medical histories (provided by the Coroner), radiology information system, pathology records and archived email communications were reviewed to obtain the demographics, dates and times of the various steps in the service process. These data were collated for each case scanned in the PMCT service.

Times were calculated in working hours and working days, excluding weekends and bank holidays, when the Coroner’s office was closed. The mean, minimum and maximum times were calculated. The reasons for delays were investigated when possible.

The purpose, time and outcome of all phone calls with families were logged, and these were reviewed for enquiries about the service, cases where families had been advised PMCT may not give the CoD, and cases where they had chosen to opt for autopsy.

All patients referred for scan through the non-suspicious death PMCT service are included in this study. Cases of overt traumatic death are part of a separate service and are not analyzed here.

These data were measured against the three standards stated above.

## Results

Between July 2015 and July 2018, 279 patients had PMCT scan, 67 (24.0%) in the family-funded service and 212 (76.0%) in the current service. The mean age in the family-funded service was 72.3 years (range 36–96), 31 male (46.3%) and 36 female (53.7%). The mean age in the LA-funded service was 72.7 years (range 37–96) 84 (39.6%) male and 128 (60.4%) female.

All cases had a whole body PMCT scan and were prepared for PMCTA and VPMCT.

### Standard 1: PMCT report to be available by day 3 from referral

**Figure 3.**
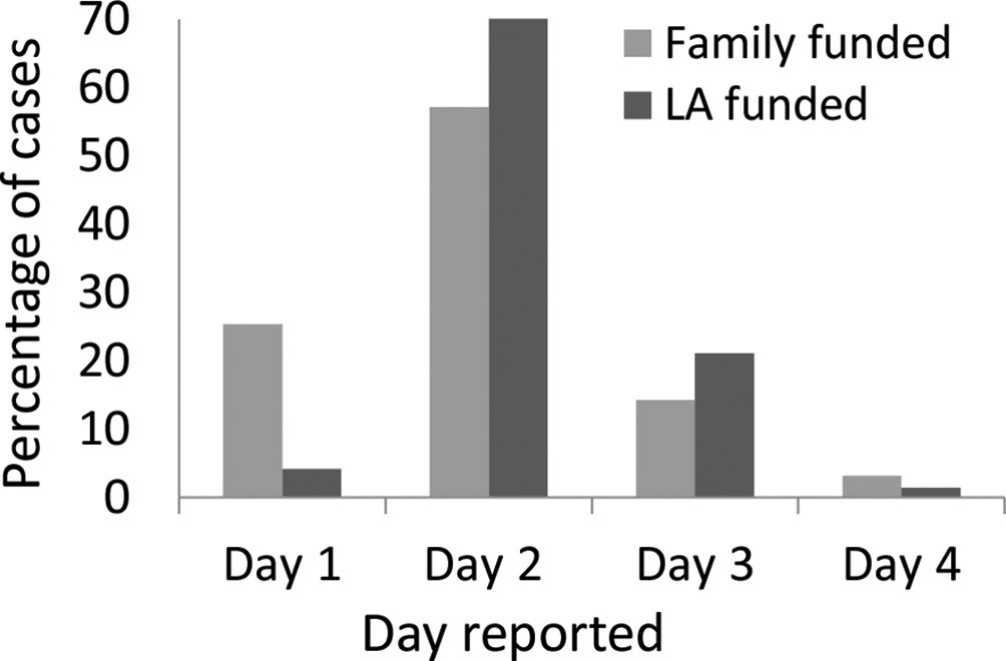
Percentage of radiology reports issued for each day from referral.

The day the scan report was issued is given in Figure 3. The time the report was issued was not available in three cases, but all these reports were issued on day 2 or 3. Families were warned of potential delays specific to scan capacity and accepted this in all cases. The mean time from referral to report was 1.65 days and 97.1% (*n* = 271/279) of cases had the radiology report issued by day 3 (96.8% *vs* 98.6% for the family-funded and LA-funded services respectively). Of the five cases reported on day 4, one report was delayed by 24 h for a second opinion from a radiologist unavailable until day 4, one was delayed due to limited scanner capacity, and the other three were due to problems with the paperwork required by the mortuary to initiate the investigation. These three cases would have been delayed for autopsy as well.

**Figure 4.**
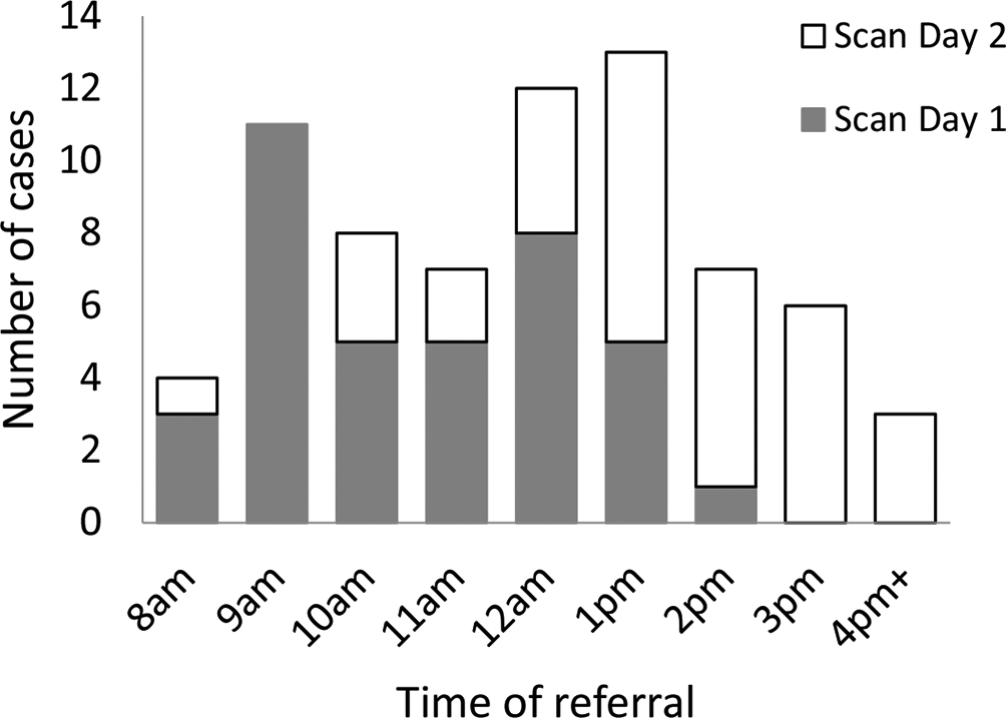
The day of scanning achieved depending on time of referral from the Coroner.

For the family-funded service, the time of referral had the greatest influence on whether the scan could be completed on the day of the referral. The median time of referral was 12:15 (range: 08:07–16:23). 58.2% of cases (39/67) were scanned on the day of referral and all but 1 of these were referred before 14:00 (Figure 4). When planning the current LA-funded service this pattern was considered, along with the triage pathologist work patterns, allowing referrals to be made or discussed by phone before midday.

Causes of delay were investigated prior to starting the current LA-funded service and addressed where possible. Increased numbers of staff, changing the process to fit with existing working patterns, addressing IT issues and in-hours scanning were all introduced to reduce delays.

### Standard 2: CoD will be given in 90% of cases

A CoD was given on the “balance of probabilities” in 97.1% of cases (*n* = 271) based on the history, external examination and scan report. The CoD was suspected on PMCT in a further two cases, but autopsy was required to screen for sepsis at the family’s request in one case and because trauma was seen on the scan that was not explained in the medical history or circumstances of death in the other. The range of causes of deaths diagnosed (Table 2) reflects those of previous studies.^2–4^

**Table 2.** Summary of causes of death and their frequency

**Region**	**Cause of death**	**Family-funded**	**%**	**LA-funded**	**%**
Cardiac	Ischaemic heart disease and its complications^a^	26	38.8%	128	60.4%
	Myocardial diseases^b^	11	16.4%	25	11.8%
Respiratory	Pneumonia	9	13.4%	17	8.0%
	Pulmonary thrombo-embolism	3	4.5%	7	3.3%
	Other	2	3.0%	5	2.4%
Vascular	Ruptured aneurysm, aortic dissection	6	9.0%	9	4.2%
Brain	Infarcts and haemorrhages	5	7.5%	8	3.8%
Gastrointestinal	Including perforation, peritonitis, pancreatitis, liver or renal failure, GI bleed	2	3.0%	8	3.8%
PMCT inconclusive/no CoD	No CoD on PMCT	2	3.0%	4	1.9%
	CoD suspected on PMCT but autopsy required	1	1.5%	1	0.5%

CoD, cause of death; PMCT, post-mortem CT.

There were more heart disease related deaths in the LA funded group (chi-square statistic with Yates correction = 0.021). This may relate to different selection of cases in the two groups or just be a statistical aberration. There was no significant difference in “inconclusive / no CoD” between the two groups.

a Complications of ischaemic heart disease include myocardial infarction and ventricular rupture

b Myocardial diseases include cardiomyopathy due to ventricular hypertrophy due to hypertensive heart disease or valve disease, and other causes of cardiomegaly

### Standard 3: less than 10% will require invasive autopsy

An invasive autopsy was required in eight patients who had inconclusive PMCT findings (2.8%) (Table 3).

**Table 3.** Details of eight patients requiring further investigation

Age/Sex	Further investigation predicted?	Why autopsy?	PMCT cause of death	Autopsy cause of death	PMCT findings correct?
39 F	Yes	Histology	Cancer	Cancer	Yes
75 F	No	Trauma seen at PMCT	Left ventricular hypertrophy	Hypertensive heart disease	Yes
67 M	No	PMCT inconclusive, no angiography or ventilation	Suspected Ischaemic heart disease	Ischaemic heart disease	Yes but uncertain
60 M	No	No cause of death on PMCT, no angiography	None	Ischaemic heart disease	No
53 F	No	No cause of death on PMCT	None	None, despite histology and toxicology	Yes
55 F	No	No cause of death on PMCT	None	None, despite histology and toxicology	Yes
65 F	No	No cause of death on PMCT	None	Tramadol and dihydrocodeine toxicity	Yes
71 M	No	PMCT inconclusive	Chest infection ischaemic heart disease	Extensive large bowel ischaemia	No

PMCT, post-mortem CT.

In one case the need for autopsy was anticipated, but the family chose to have the PMCT to minimize the extent of the autopsy.

In one case trauma (chest wall injury with surgical emphysema, pneumothorax and acromioclavicular subluxation) was found on the scan that was not explained in the circumstances of death. The PMCT observation of left ventricular hypertrophy was not considered a sufficient cause of death in this circumstance. At autopsy, no other significant pathology was discovered, and the trauma was attributed to the patient falling as death occurred, in keeping with the position in which they were found. This had not been appreciated during the initial external examination as livor mortis had masked the findings.

In one case moderately diseased but patent coronary vessels on PMCT were considered inconclusive for the suspected diagnosis of ischaemic heart disease. Full autopsy also showed moderate coronary artery disease and no other cause of death, and therefore this was given as the cause of death on the “balance of probabilities.”

Two cases were hampered by not being able to complete targeted coronary angiography reducing the diagnostic confidence of the study.

In two cases, the CoD was unascertained after autopsy, histology and toxicology. At inquest the causes of death were amended to sudden cardiac death with morphologically normal heart. In one case, there was a history of diarrhoea and vomiting a week prior to death and it was postulated an electrolyte imbalance may have led to a fatal arrhythmia. In one further case autopsy was normal, and death was due to drug toxicity. The findings of the PMCT scans therefore agreed with autopsy in these three cases.

## Cannulation and intubation

Failed angiography is generally due to difficulties inserting the angiography catheter for the PMCTA. This is performed via a cut-down insertion into the left carotid artery, passage of a 16 Fr Foley catheter into the ascending aorta and inflation of a 30 ml balloon with diluted contrast to create a seal.^6^

Successful cannulation (tip in the ascending aorta) occurred in 49 cases (75.4%) in the family-funded service compared to 191 cases (90.0%) in the current LA-funded service.

Overall 37/279 cases had cannulation problems: the failure rate reduced from 24.6% in the family-funded service to 10.0% in the LA-funded service. 8 cases (3%) had failed cannulation (the catheter was in a vein or not in a vessel), 11 cases (4%) had mis-sited catheter due to pathology (haemopericardium or calcified atheroma in the carotid artery or aortic arch) and in 18 cases (7%) the catheter passed in to the descending rather than ascending aorta. The failure rate improved as experience grew and techniques were developed to enable PMCTA to be completed when the catheter was in a suboptimal position. If the catheter is in the descending aorta, re-siting the catheter is attempted after the balloon is partially deflated. If the catheter cannot be repositioned in the ascending aorta, the tip of catheter is positioned in the arch of aorta and the balloon left deflated, allowing both negative and positive contrast flow into both the descending and ascending aorta, delineating the coronary arteries. If there is concern about pathology in the major vessels causing cannulation problems, *e.g.* haemopericardium from aortic dissection, hand injections of contrast are used first to avoid exacerbating the damage.^6,7^

53 (79.1%) of the family-funded cases and 203 (95.8%) of the current service cases had VPMCT scans. Where there was an existing endotracheal tube this was used. Failure of VPMCT was generally due to an incorrectly sited tube. 2 of the 12 failed ventilation cases in the family-funded service required autopsy (Table 3), but in these cases failure to give a cause of death was attributed to the failure of angiography. The remaining unsuccessful ventilation cases were given cardiac or vascular causes of death. None of the eight current service cases with failed VPMCT required autopsy, five had cardiac CoD, one had intracerebral haemorrhage and two had pneumonia.

### Service enquiries that did not proceed to scan

During the original family-funded PMCT service there were 46 enquiries which did not go on to have a scan. In 25 of these cases, a pathologist advised that further tests would be required (Table 4). Where PMCT alone was considered likely to give a cause of death, the main reason families decided not to proceed was that locally the PMCT service could not guarantee turn around at the same speed as autopsy. In two cases, the pathologist decided they could issue a CoD immediately without either PMCT or autopsy on the balance of probabilities based on information already available.

**Table 4.** Reasons families were advised PMCT may not provide the CoD and the reasons families gave for not continuing with PMCT

**Pathologist states PMCT may not be sufficient**	**Frequency**	**Family decided not to proceed**	**Frequency**
Toxicology or histology required	10	Possible delay to get CoD	12
PMCT not expected to give CoD	4	Cost	3
Concerns about care	8	Reason unknown	4
Possible unnatural death	1		
Advanced decomposition	1	**PMCT not required**	
Body habitus too large for scanner	1	CoD given from records and external	2

CoD, cause of death; PMCT, post-mortem CT.

### Feedback

The goal of this service was to improve the experience for the families. To achieve this, the service had to meet the needs of all service users, including the families, Coroner’s Officers and undertakers. Formal sequential feedback was not requested from families by the coroner’s officers and is therefore not available. Despite this, all coroner’s officers reported consistently positive feedback from families. Some examples of the feedback received:

" …most importantly gives the community and bereaved families an alternative to the standard invasive autopsy…..removes much of the distress experienced by families during a very difficult time" from an Undertaker.

"In conversation with colleagues further afield it only highlights that this process has set Leicester apart as a leader in its field and the need to make this a standard service across the country" the President, Midlands Area Federation, National Association Funeral Directors.

"one gentleman whose wife had a PMCT was so relieved and pleased we had not ‘…hurt his wife’. He gave me his sincerest thanks and gratitude for scanning her. He sobbed with relief down the phone when I told him." From a Coroner’s officer.

## Discussion

The service achieved the day 3 target in 97.1% of cases, with the other scans being reported on day 4. As the service has grown and become local authority funded, processes have become more efficient due to not having to arrange funding from families and operating a post-mortem specific CT scanner session. Increasing the number of radiologists trained to report scans and changing the triage system to enable pathologists to vet cases in the morning when they were timetabled to be in the mortuary has also improved efficiency.

One person each day was responsible for co-ordination of the service and occasionally other duties took priority causing delays of up to 2 h in 11 cases. The infrequency and unpredictability of the family-funded service exacerbated this. Adapting the process with referrals being made in the morning for the LA-funded service and consistently scanning more cases meant priority could be given to this work by the administration staff. This factor also improved the efficiency of the mortuary process and communication between the teams.

Considerably less than 10% of cases required an autopsy, achieving our standard. These findings are consistent with published studies suggesting that PMCT with VPMCT and PMCTA can replace approximately 90% of invasive autopsies.^2,3^

As angiography has been shown to increase the accuracy of PMCT,^2,5^ failure to perform PMCTA reduced confidence and contributed to two autopsies being required. Problems inserting the angiography catheter have been reported^6^ and decrease with experience. It has been recognized that cannulation is more problematic in patients with short necks and those with rigor^6^ and with some pathologies^2,5^ irrespective of who (pathologist or mortuary technician) has done the cannulation.

The predicted need for further tests, such as toxicology, microbiology or histology was the most common reason to triage away from PMCT. Although toxicology could be obtained prior to PMCT, the rationale for avoiding these cases was that the delay in the result could mean that autopsy would be considerably delayed if ultimately required, or the body inappropriately released if toxicology subsequently shows the need for further tests. Quicker sample processing time would resolve this issue.

For some families being told the cause of death may not be available until day 4, caused them to opt for an autopsy for a faster service. Although most cases received a cause of death on day 2, we felt it appropriate to stick with the day four guarantee rather than a day two aspiration. However, some centres providing the Coroner’s autopsy work struggle to deliver autopsies within this time frame and providing a PMCT report and cause of death by day three would be considered rapid.

It is acknowledged some natural pathology will be missed on PMCT, even with angiography and ventilation. As these cases are assessed on the “balance of probabilities”—the legal standard required for Coronial investigation—a degree of uncertainty is considered acceptable. This should be known and acknowledged by all involved in using PMCT to provide a cause of death. If there is significant doubt in the diagnosis, or the differential diagnosis of the imaging findings include unsuspected unnatural death, an autopsy is generally performed.

In addition to the limitations of the service, there are limitations to this analysis. Some data were not collected at the time it occurred and were irretrievable. This has now been addressed. We also recognize that this “scan only” approach may be less relevant in countries where legislation is different, or autopsy rates are lower. However, the principles of the service may be appropriate to any death investigation, whether from natural causes or otherwise.

This paper demonstrates that a PMCT service can be set up and successfully run within a national health service without impacting upon clinical services, as long as sufficient equipment and staff are made available. This allows us to run this service as part of an ongoing responsibility to patient care.

### Conclusion

The service exceeded set standards and met the needs of the Coroner and the families based on the feedback received. A service based on LA funding with a higher number being triaged to PMCT is more efficient to run with reduced times and cost, allowing a PMCT service to run within the framework of mortuary funding. This model could be employed for similar services, but the change to the logistics and financial structures required to initiate such services remains a significant hurdle, even if long-term costs remain the same.
